# Protective Role of False Tendon in Subjects with Left Bundle Branch Block: A Virtual Population Study

**DOI:** 10.1371/journal.pone.0146477

**Published:** 2016-01-14

**Authors:** Matthias Lange, Luigi Yuri Di Marco, Karim Lekadir, Toni Lassila, Alejandro F. Frangi

**Affiliations:** 1 Center for Computational Imaging and Simulation Technologies in Biomedicine, The University of Sheffield, Sheffield, United Kingdom; 2 Center for Computational Imaging and Simulation Technologies in Biomedicine, Universitat Pompeu Fabra, Barcelona, Spain; University of Oxford, UNITED KINGDOM

## Abstract

False tendons (FTs) are fibrous or fibromuscular bands that can be found in both the normal and abnormal human heart in various anatomical forms depending on their attachment points, tissue types, and geometrical properties. While FTs are widely considered to affect the function of the heart, their specific roles remain largely unclear and unexplored. In this paper, we present an *in silico* study of the ventricular activation time of the human heart in the presence of FTs. This study presents the first computational model of the human heart that includes a FT, Purkinje network, and papillary muscles. Based on this model, we perform simulations to investigate the effect of different types of FTs on hearts with the electrical conduction abnormality of a left bundle branch block (LBBB). We employ a virtual population of 70 human hearts derived from a statistical atlas, and run a total of 560 simulations to assess ventricular activation time with different FT configurations. The obtained results indicate that, in the presence of a LBBB, the FT reduces the total activation time that is abnormally augmented due to a branch block, to such an extent that surgical implant of cardiac resynchronisation devices might not be recommended by international guidelines. Specifically, the simulation results show that FTs reduce the QRS duration at least 10 ms in 80% of hearts, and up to 45 ms for FTs connecting to the ventricular free wall, suggesting a significant reduction of cardiovascular mortality risk. In further simulation studies we show the reduction in the QRS duration is more sensitive to the shape of the heart then the size of the heart or the exact location of the FT. Finally, the model suggests that FTs may contribute to reducing the activation time difference between the left and right ventricles from 12 ms to 4 ms. We conclude that FTs may provide an alternative conduction pathway that compensates for the propagation delay caused by the LBBB. Further investigation is needed to quantify the clinical impact of FTs on cardiovascular mortality risk.

## Introduction

False tendons (FTs) are additional fibrous or fibromuscular strings occasionally located inside the ventricles, and which are attached to either the ventricular wall, the septum, or the papillary muscles (PMs) [1]. Autopsy investigations have shown a high prevalence of these anomalies, ranging from 34% to 68% of the population (Table 1) in healthy humans. A wider prevalence range is reported in echocardiographic studies, from less than 1% to more than 85% (Table 1), which shows the difficulty in imaging FTs *in vivo*, which in turn makes it difficult to study their significance and possible effects on the cardiac function.

**Table 1:** Studies of the Prevalence of False Tendons (FTs) in the Human Heart by Autopsy (a) and Echocardiography (b).

	Reference	Study size	FT prevalence (%)
(a)	Gerlis et al. [21]	686	48
	Luetmer et al. [22]	483	55
	Boyd et al. [23]	474	68
	Abdulla et al. [3]	100	34
	Grzybiak et al. [24]	180	40
	Kervancioğlu et al. [1]	8	63
(b)	Okamoto et al. [25]	132	46
	Nishimura et al. [26]	1000	<1
	Perry et al. [27]	3847	<1
	Suwa et al. [5]	1117	6
	Sethuraman et al. [28]	1012	<1
	Brenner et al. [29]	100	61
	Vered et al. [30]	2079	2
	Malouf et al. [31]	488	25
	Casta and Wolf [32]	218	14
	Suwa et al. [6]	187	71
	Cangelosi et al. [33]	916	26
	Cocchieri and Bardelli [34]	273	29
	Kervancioğlu [1]	368	26
	Lie et al. [35]	99	85

Autopsy studies show that FTs comprise different tissue types including connective, conductive, and fibrous tissues, as well as blood vessels [1–5], which suggests that their presence is likely to intervene in the cardiac electrical conduction and more generally in the function of the heart. This is further supported by reports of isolated premature ventricular contractions [5–7], electrical activation re-entry [4], tachycardia [8, 9], and electrocardiographic changes [10–14] in subject with FTs. So far, due to the complexity of the FTs and the challenges associated with their *in vivo* imaging, their exact significance, effects and possible risks to the human heart remain largely unclear [4, 15, 16].

On the other hand, despite their relatively high prevalence and the increasing clinical interest associated with FTs, to the best of our knowledge there exists in the literature no computational model assessing the activation times of the human heart that includes the influence of FTs. Yet, such a model would constitute a valuable complement of clinically-motivated studies for simulating and understanding the effects of FTs on specific aspects of the cardiac function such as electrical conduction. Furthermore, it would allow the study of the role of FTs under a wide range of scenarios through the use of virtual populations and numerical simulations, both in the healthy heart and also in relation to specific cardiac anomalies, such as the left bundle branch block (LBBB), which has been observed simultaneously with a FT in a recent case study [14].

LBBB is a condition affecting 1–2% of the general population [17], for whom the activation of the left ventricle is delayed due to the disruption of the electrical conduction along the left bundle branch. In the presence of a complete LBBB, the left ventricle is activated by the electrical stimulus propagating from the right ventricle. Thus, the overall ventricular activation time increases, as reflected by the widening of the QRS complex in the surface electrocardiogram (ECG).

As the risk of cardiovascular (CV) mortality increases for QRS lengths above 80 ms [18], patients suffering from this condition may benefit from cardiac resynchronisation therapy (CRT). However, the installation of CRT devices carries inherent risks, and international clinical guidelines [19, 20] indicate an empirical threshold (QRS_d,TH_ = 120 ms) of the QRS duration (QRS_d_), below which CRT is not recommended. On the other hand, FT could in principle reduce QRS_d_ and thus the risk of CV mortality.

Consequently, the objective of this paper is twofold. First, we propose to the best of our knowledge the first computational model of the human heart that includes a FT. Subsequently, we use our model to test the hypothesis that the presence of FT in the left ventricle reduces the LBBB-induced QRS prolongation. The reduction is then quantified with respect to the CV mortality risk based on [18], in particular whether the QRS_d_ falls below QRS_d,TH_.

To this end, we address the following key technical challenges: 1) We derive a representative virtual population of ventricular models, within which different configurations of FTs are generated. 2) We introduce a computational approach for modelling the PMs, which is a critical step in this process as FTs are in some configurations connected to the PMs. 3) We extend the existing literature on the Purkinje (PK) system to incorporate both the PMs and FTs. 4) Finally, we generate several types of FTs based on the existing literature from autopsies and histological studies. To validate our model, we perform a total of 560 simulations of the ventricular activation time to assess the action potential (AP) propagation under different FT configurations and in the presence or absence of bundle branch blocks.

## 1 Methods

The proposed computational model requires the modelling of various structures and elements that are involved in the definition and electrical function of FTs. These include the ventricles, within which the FTs are located. In this *in silico* study, a virtual population of 70 ventricles is generated from a statistical cardiac atlas, as detailed in Section 1.1. Next, we enrich the computational model, as described in Section 1.6, with PMs as they can serve as connection points for the FTs. Additionally, due to its importance in electrical conduction, a new PK system that takes into account the presence of PMs is generated in Section 1.3 The modelling of the FTs themselves is presented in Section 1.4. Finally, using the solver and protocol detailed in Section 1.6, we perform simulations of the ventricular activation time based on the introduced computational model and the virtual population of ventricles.

### 1.1 Ventricular model

To simulate the effect of FTs on the human heart, a sample of hearts with variable ventricular geometries is required. Previous simulation works have used simplified geometries such as an ellipsoidal model [36] of the left ventricle (LV) or have acquired animal models [37–39], which can differ from the human heart in many aspects. More recently, patient-specific simulations of full-heart human electrophysiology have emerged [40]. Instead, in this paper we use a virtual human heart population generated from a statistical atlas, which accounts for differences in the shape and size of the ventricles, but without linking the hearts into any specific patient instances.

More specifically, the *in silico* population of human ventricles produced in this study is based on the statistical cardiac atlas by Hoogendoorn et al. [41], which was built from 134 real patients based on high resolution CT image data, which resulted in high quality and realistic surface meshes of the cardiac structures. Such an atlas is typically constructed from a representative sample of human subjects and provides an average shape of the anatomy, together with the main axes of deviations from this average. Mathematically, each shape can be described using the following equation:

$$\begin{array}{c} x=\bar{x}+Pb, \end{array}$$ (1)

where **x** is a vector of size 3*n* representing the shape in terms of its *n* 3D landmark points. Here $\bar{x}$ corresponds to the mean shape of the model and **P** is a *t* × 3*n* matrix that encapsulates *t* eigenvectors describing the main directions of variation in the model. Each unit vector is associated with an eigenvalue λ_*i*_, *i* ∈ {1, ⋯, *t*} that describes the amount of allowed variation along each axis. Finally, **b** = (*b*_1_, ⋯,*b*_*t*_)^*T*^ is a vector that encapsulates the *b*_*i*_ weights that control the deviation of the shape **x** from the mean $\bar{x}$.

In this work we chose *t* = 10 modes of variation to generate the virtual shapes, by randomly varying the deviation weights *b*_*i*_ within the allowable bounds of the model $-3\sqrt{\lambda_{i}}\leq b_{i}\leq +3\sqrt{\lambda_{i}}$ [42]. A virtual population of 70 ventricular meshes were generated and used to run a large number of AP propagation simulations based on varying configurations of the PMs, FTs, and the PK system, as detailed in subsequent sections.

### 1.2 The papillary muscle

The PMs are common endpoints for the FTs and therefore they must be modeled so that the computational model has the capability to represent such configurations. However, due to the complexity of PMs, *in silico* models of the heart usually do not include PMs (nor the trabeculae). In this work, we propose a statistical approach to PM modeling. More specifically, seven CT datasets were acquired and used to assess key anatomical features of the PM [43–45]. These include length, diameter, attachment points, angle inclination from base to tip, and distance to the LV (more details can be found in Table 2). Subsequently, the statistical variability of these parameters was estimated, thus allowing to generate in new ventricular models virtual PMs with multiple configurations and varying properties in new ventricular models. Note that only subjects with a single posterior papillary muscle (PPM) and a single anterior papillary muscle (APM) are included in the sample. This is sufficient for the experiments with the FT, as majority of FTs connect to one PM [4]. Furthermore, the APM is in most cases singular [46].

**Table 2:** Statistical Parameters of the Anterior Papillary Muscle (APM) and the Posterior Papillary Muscle (PPM), Calculated from Landmarks in the Left Ventricular (LV) Computer Tomography Scans.

Parameter	Landmark relation	APM	PPM
LV axis	A line connecting the LV apex and the center of mitral valve.	N/A	
LV length in mm	Length of the LV axis.	98.0 ± 8.5	
Normalised^†^ PM diameter	Distance between landmark ^(3)^ and ^(4)^.	8.9 ± 1.7	8.3 ± 1.9
Normalised^†^ PM length	Distance between the attachment point^(1)^ and tip^(2)^.	33.6 ± 5.3	27.6 ± 5.5
PM attachment triangle	The triangle in the LV-Mesh that is closest the attachment point^(1)^.	N/A	N/A
Angle in degree between PM tip and attachment point	Angle between the line from the attachment point^(1)^ to the LV axis and the line from the tip^(2)^ to the LV axis in a plane perpendicular to the LV axis.	19.9 ± 6.3	27.8 ± 12.2
PM tip distance in mm from LV	A line starting from the LV axis and perpendicular to it through the tip point and intersection the LV-Mesh.	24.1 ± 3.1	21.7 ± 3.8

^(i)^ Landmark (i) in Fig 1, for i = {1,2,3,4}

N/A: Not applicable

^†^normalised with respect to LV length and multiplied by 100

The CT image data were acquired from patients who underwent a CT examination as part of their routine diagnostic protocol for suspected coronary artery disease. The CT scanner used for this purpose was a 64-row detector Toshiba Aquilion 64 system (Toshiba Medical Systems, Tochigi, Japan), with contrast material of 80 to 100ml Xenetic 350 applied at a rate of 5ml/s, which allowed to enhance the appearance of the PMs. The obtained resolution of the images was of 0.4 mm × 0.4 mm × 2.0 mm.

**Fig 1:**
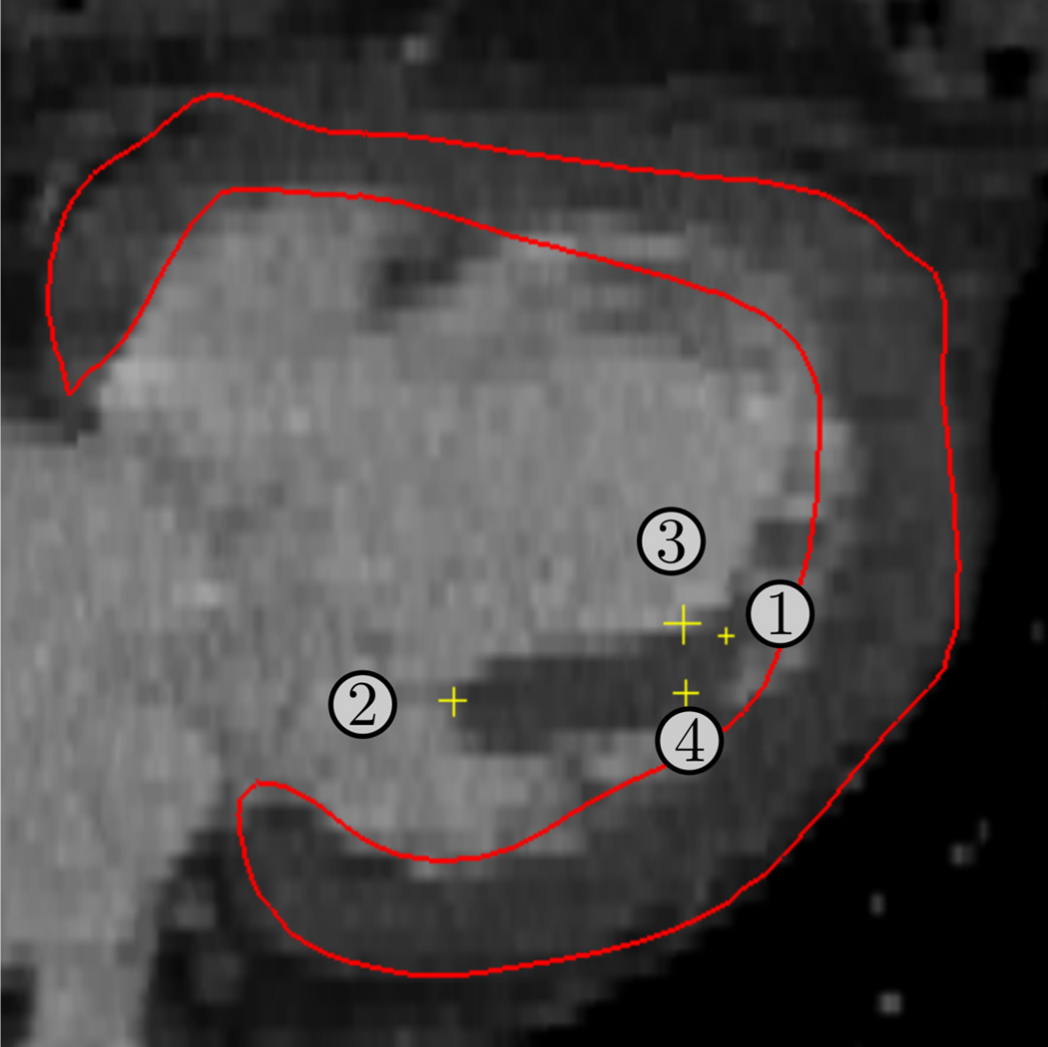
A computed tomography slice and mesh of the left ventricle (red) with the landmarks (yellow). This has been used to build the statistic of the papillary muscle: (1) Attachment point (2) Tip of papillary muscle, (3–4) Landmarks for the diameter estimation.

To obtain the length, diameter and direction from the base to the tip of the PM (referred to as orientation), key anatomical landmarks were consistently defined on the CT images by using the medical image analysis software GIMIAS v1.5 (www.gimias.org). More specifically, four landmarks were selected manually as the center point of the area where the PM joins the LV (*i.e*., attachment point), the tip of the PM and two landmarks that determine the diameter of the PM (Fig 1). Two further quantities were automatically calculated from a LV atlas instance, which were fitted to the image, *i.e*. the center of the mitral valve and the left ventricular apex. Table 2 shows the relationships between the landmarks and the PM parameters, together with the mean and standard deviation values.

We represent a PM model in this work by combining a cylinder and a paraboloid, where this choice of shape is based on images of PM found in the literature [46–48]. The parameters for the cylinder and paraboloid are indicated in Table 2. The cylinder is defined as 60% of the PM length. The remaining part of the PM is represented by the paraboloid given in cylinder coordinates with origin at the center of the bottom of the cylinder *z*(*r*, *φ*) = *L* − 4 ⋅ 0.4*Lr*^2^/(*d*^2^) where *d* is the diameter of the resulting PM, *L* is the length, 0.4*L* is the height of the paraboloid, and *r* is the radial direction.

**Fig 2:**
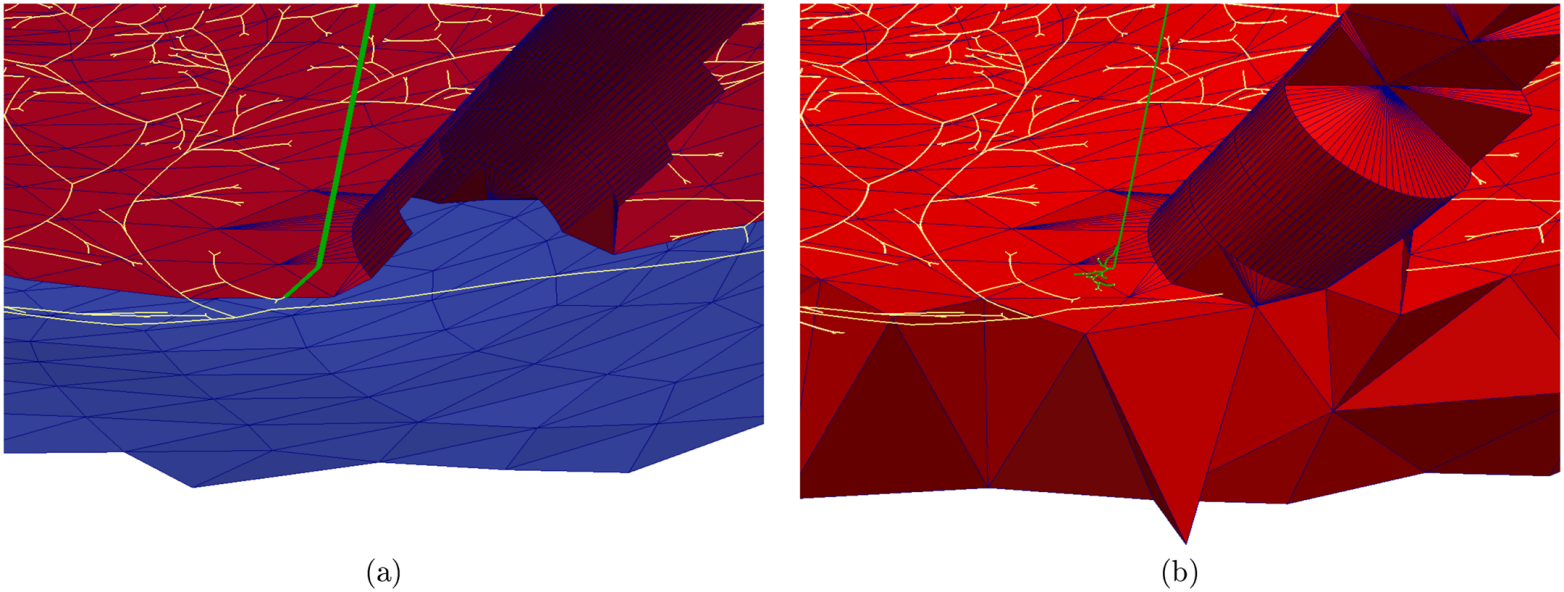
Different terminations of the false tendon (green) with Purkinje (PK) fibre system (white). (a) False tendon directly connected to the main PK system, on a left ventricular surface based on triangles with papillary muscle, where the endocardium is red and the epicardium is blue. (b) False tendon terminating in small PK fibre branching, on a left ventricular volume mesh based on tetrahedra with papillary muscle, with myocardium in red.

The PM is then placed at one of the statistically defined attachment points, oriented according to the statistics, and merged with the ventricular model, which results in a new LV surface with a PM as illustrated in Fig 2.

### 1.3 Purkinje fibre generation

The coordinated and fast activation of the heart is ensured by an insulated network of fast conduction cells, which span the endocardium. The so-called PK network, which delivers the Action Potential (AP) to the ventricles. The pattern in which the AP travels the ventricle is critical to the efficiency of the heart contraction and, therefore, it is important to consider the PK network in our simulation. However, thus far, the *in vivo* imaging of the PK fibre network remains infeasible. This means its construction in computational models of the heart is usually achieved manually [49], with rule-based deterministic models [50, 51], or extracted from *ex vivo* images [52]. However, existing techniques do not take into account the presence of PMs and FTs. In this paper, we propose an extension of the algorithm of Sebastian et al. [51] to account for these additional structures, as well as to adapt it for the right ventricle (RV).

The PK network is algorithmically generated with an L-system. This iterative process uses two basic elements, *i.e*., L- or Y-shaped structures, which are generated progressively from a given starting point. For the Y-structures, the direction of growth is from the lower part to the upper part, which then creates two new starting points. In L-structures, the growth starts at the lower left corner and advances toward the two endpoints, which in turn creates new starting points. At the next iteration, a new starting point is randomly selected from the set of starting points. With this implementation the resulting network depends on the angle of the L- or Y-structure, the length of the branches, and the number of points from which each of the segments are built, because they allow structure flexibility and thus the newly grown fibres can avoid the already existing network. More details can be found in [51].

**Fig 3:**
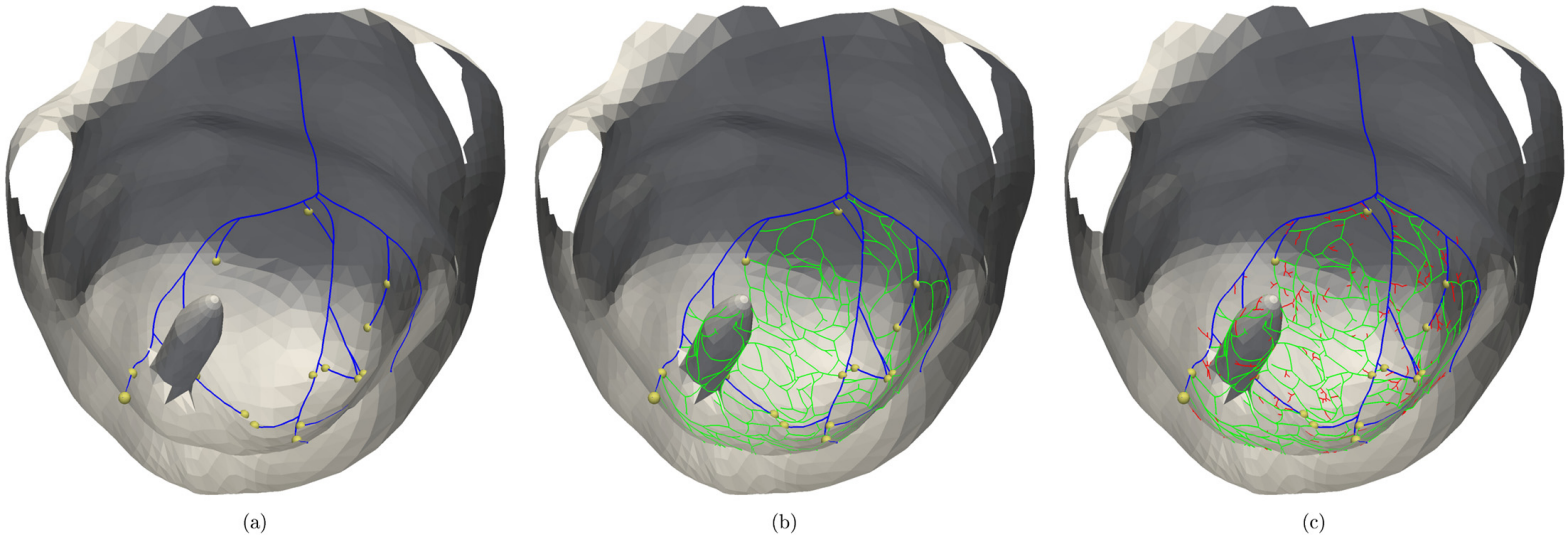
The three stages of the Purkinje growing algorithm. Algorithm applied on the left ventricular surface with papillary muscle. (a) First stage with main fibres in blue and endpoints marked by yellow sphere, (b) finer second stage in green and (c) with final branching of the last stage in red.

Our modified algorithm for the generation of a PK system in the LV consists of three stages, where the first is landmark driven and the last two are deterministic rule-based. In the first stage the left bundle branch and the three main branches are created (Fig 3a). To do so a Purkinje fibre is generated from the starting point of the left bundle branch in the direction of the ventricular apex, and branches after two-thirds of the way in the three main fibres. Two of these fibres proceed towards the PPM and APM, respectively, and the third towards the apex. After reaching their landmarks all fibres are grown towards the base of the heart, where they stop 2 cm before reaching the basal plane. After the three main fibres have been grown with an L-rule, a deterministic fibre generation at the endpoints is started with an Y-rule. Note that all the branches are grown underneath the PM (Fig 2a) and continued on the endocardial surface after passing the PM. The second stage (Fig 3b) creates new Y-structures at the endpoints of the developing PK graph, which extend onto the PM. The last two stages create a homogeneous network with loops for redundancy, while the final stage (Fig 3c) increases the density of Purkinje muscle junctions, without creating looping PK fibres. Therefore, based on the American Heart Association segment model [53], random points are selected from the previous PK network and used as possible starting points. At some of these points smaller Y-structures are generated.

For the RV, the bundle is grown from the AV-Node towards the apex of the RV. Again at two-thirds of the way the branch splits in two, where one branch progresses to the apex and the other crosses to the right ventricular wall [54]. The second and third stages are essentially the same as for the LV, and differing only in stage two, which begins with only two starting points and in which the fibres do not avoid each other.

The growth parameters for the LV and RV are summarised in Table 3.

**Table 3:** Values used for the Deterministic Purkinje Network Growing in the Left Ventricle (LV) [51] and Right Ventricle (RV).

	Second step		Third step	
	LV	RV	LV	RV
Branch Length [mm]	6 ± 0.3	4 ± 0.3	1 ± 0.03	1 ± 0.03
Branch angle [degree]	60 ± 40	60 ± 20	60 ± 40	60 ± 40
Number of Segments	10	10	5	5
Max. branches	300	800	600	1000

### 1.4 Creation of false tendons

After generating the PK network, as detailed above, the main contribution of this work, the FT, is finally incorporated to the computational model of the electrical conduction system of the heart.

The FT model is based on clinical observations that 60% of FTs feature conductive tissue [4] and this tissue shows the same microscopic structure as the bundle of His. Moreover, the fibres are connected to the bundle of His [3, 4, 55], which suggests that the bundle of His radiates into the FTs [3, 4]. For this reason, we model the FTs as additional PK fibres, which are attached to either the ventricular wall, the septum, or the PMs. We made this simplification because the fast conduction tissue is the only tissue that influences the total activation time, *i.e*., the time elapsing while the AP propagates from the AV node to the last activated muscle cell.

We begin the modelling process by automatically selecting the starting point of the FT at the left bundle branch, and by selecting the endpoint on the APM, PPM, or the ventricular free wall, depending on the desired FT configuration. Due to the lack of knowledge on how the Purkinje fibre in the FT merges into the PM or ventricular muscle, we distinguish two different connection types at the endpoint. The first one is a direct connection of the PK fibre in the FT to the PK system on the LV, which is constructed by an additional PK fibre segment that connects the endpoint of the FT to the closest PK fibre point (Fig 2a). The second case is a delta connection, which branches from the endpoint of the FT into a tree of PK fibres. This is achieved with the PK growing algorithm starting at the endpoint with a segment length of 0.2 ± 0.1 mm and a branching angle of 60.0 ± 1.0°. The algorithm generates ten branches, which do not connect to the main PK system. (Fig 2b).

### 1.5 Modeling of a left bundle branch block

In this study we investigate the influence of FTs on hearts with conduction disturbances. Since the FT provides an additional conduction path from the bundle of His to the PK network, it is important to understand its benefit in the presence of a bundle branch block.

We model the LBBB in our PK fibre network by assuming that the activation of the FT is not affected, which means the LBBB occurs downstream of the FT insertion point. This assumption is supported by the reported origin of the FT from the bundle of His and a recent publication by Irie et al. [14], who report a case of LBBB in the presence of a FT. In that particular case the FT is connected to the ventricular free wall and it is reported that ‘the lateral LV wall contracted during systole and relaxed during diastole, whereas the septum expanded during systole and contracted during diastole’ Irie et al. [14]. This behaviour can originate from an AP traveling through the FT and activating the ventricular free wall.

The chosen region for the LBBB is assigned a flag in the computational model, such that in the AP propagation simulation we can set the conduction velocity in that particular region to zero. This corresponds to a full conduction block.

### 1.6 Ventricular activation time estimation

In the second part of the paper (validation section), we illustrate the potential of the proposed computational model to study the effects of FTs in the electrical activation of healthy hearts and in hearts with a LBBB. To this end, the AP propagation simulations need to be carried out, for which different modelling approaches exist. For the intended experiments an estimate of the ventricular activation time is need, while the ionic currents over the cell membrane will not be altered or be of interest. For this reason, we choose the Eikonal equation to model the AP propagation. This describes the wave propagation according to a prescribed constant conduction velocity without modelling the cell membrane. More importantly, it has been shown that the Eikonal approximation gives good approximation of the myocardial activation time [56–58] needed for our experiments. There also exist more sophisticated models, such as the bidomain model [59–62], which models the electrical activity in- and outside of the cell, and over the cell membrane. A simpler model is the monodomain model [52, 59–62], which assumes equal anisotropy ratios between the intra- and extracellular conductivities, but uses the same cell membrane modelling methodology. The monodomain and bidomain models are diffusion reaction models, which are usually solved with a spatial discretisation method, such as finite volumes or finite elements [63], on a high resolution volume mesh (1 000 000 vertices or more). This makes the models computationally very demanding, and unsuitable for the large number of simulations we intend to run.

The following Eikonal problem is solved with the fast marching method [64]

$$\begin{array}{c} \left\{ \begin{array}{cc} \nabla \phi \text{AF}{\left(\text{AF}\right)}^{T}\nabla \phi^{T}-1=0 & x_{i}\in \Omega \\ \phi \left(x_{i}\right)=\phi_{0}\left(x_{i}\right) & x_{i}\in \partial \Omega , \end{array}\right. \end{array}$$ (2)

where *Ω* is the problem domain, *ϕ*_0_ are the known activation times and *ϕ* is the activation time to be obtained, the orthogonal matrix **A** is composed from the unit vectors pointing along the myocardial fibre orientation and **F** represents the conduction velocity on the diagonal.

**Fig 4:**
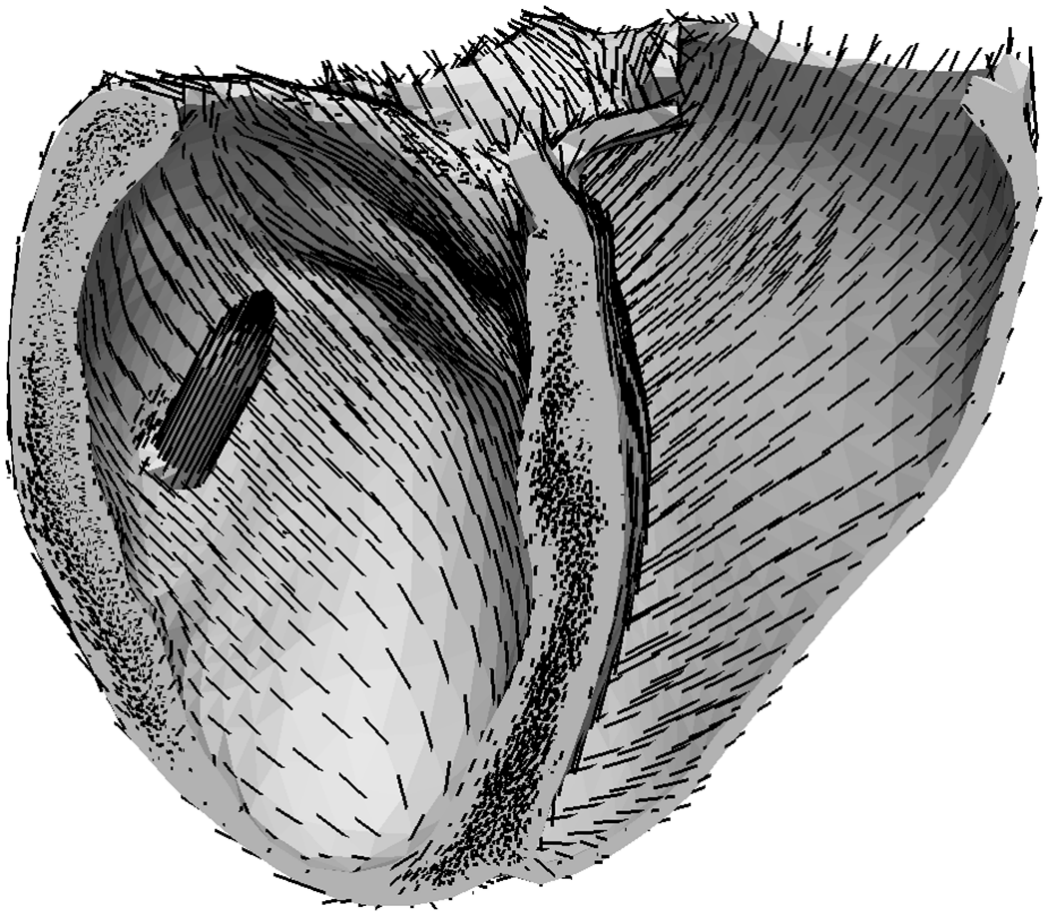
Example of the myocaridal fibre orientation. Myocardial fibre orientation generated with the Streeter model in one exemplary heart.

For the simulation, tetrahedral volumetric meshes with approximately 70 000 vertices (Fig 2b) are generated from the ventricular surfaces with PMs using the TetGen library. For all vertices of the tetrahedral mesh, the myocardial fiber orientation is estimated using the Streeter model [65] with a linear interpolation between endocardium and epicardium. While for the RV and LV the same rules are applied for vertices in the PM the fiber orientation is aligned to the long axis of the PM as described in [66, 67] (Fig 4). At the border between ventricle and PM the fibres are locally smoothed by the solver as it uses the average fibre orientation per tetrahedra.

The volumetric mesh together with the one dimensional PK system provides the spatial domains *Ω* within which the Eikonal equation is solved for the estimation of the activation time of the heart. Both domains are coupled at the endpoints of the PK network, where the AP propagation from the PK system to the myocardial system is delayed by 6 ms and 1 ms in the opposite direction [68, 69]. Here the PK fibre system includes the FT, such that there is no additional delay between the normal PK fibres and the FT fibre. In the case of a delta ending only, which connects the FT to the myocardium, we apply a delay of 6 ms.

The Eikonal equation is solved with the fast marching method proposed in [64], where the myocardial conduction velocity is equal to 0.8 m/s in the longitudinal direction and to 0.3 m/s in the transversal direction. For the PK fibre network and for the FT, we set the conduction velocity to 3.5 m/s.

## 2 Virtual population experiments and results

### 2.1 Sensitivity of the QRS_d_ to different Purkinje network topologies

Before evaluating the computational model of the FTs, we estimated the variability of the QRS_d_ due solely to different configurations of the PK system. To achieve this, we chose an arbitrary ventricular mesh with PMs and generated the volumetric mesh with fibre orientations. To this ventricular model we then applied our PK growing algorithm (Sec. 1.3) with the parameters from Table 3, *i.e*., for a total of 25 different PK systems. Two parameters (length, branching angle) were varied following a Gaussian distribution (Table 3), while the remaining eight parameters were fixed. For each of the PK systems, the Eikonal equation was solved with the same ventricular geometry and then we estimated the myocardial activation time. From the myocardial activation time the QRS_d_ was calculated as the time difference between the first myocardial activation and the time at which the entire myocardium has been activated. The electrical charge in the PK network is negligible and will not be captured by a surface ECG [70, 71]. We obtained a mean QRS_d_ of 108 ± 6 ms with a range from minimal 94 ms to maximal 118 ms. For the aforementioned simulations the model did not include FTs.

### 2.2 Influence of false tendons on the ventricular activation time

**Fig 5:**
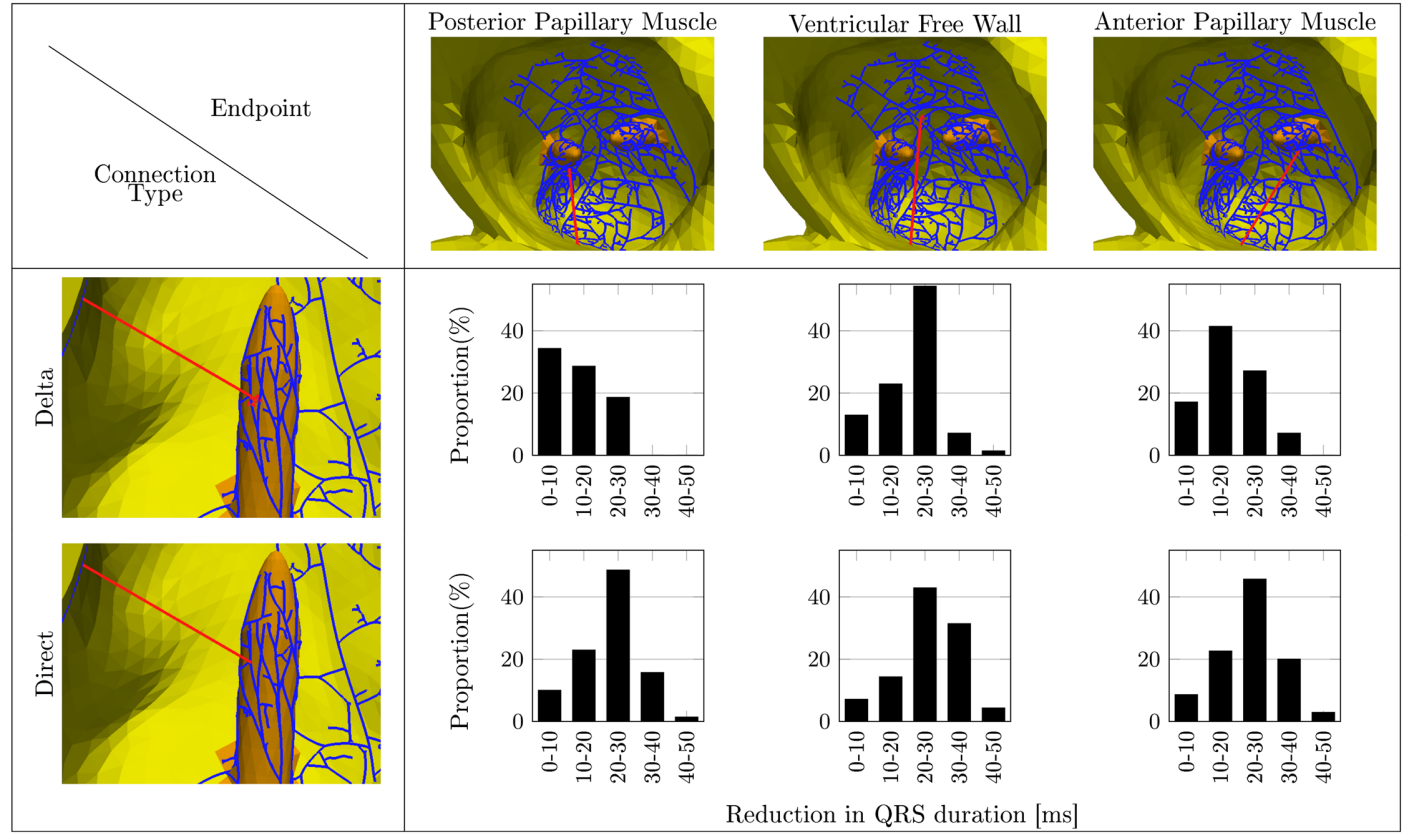
Shortening of the QRS grouped by decades of shortening. Shortening given for the direct and delta connected false tendon to various endpoints. The mean QRS duration without FT was 131.9 ± 7.1 ms.

The aim of the following experiment was to investigate the influence of different FTs configurations on the QRS_d_. To do so, we conducted simulations with 560 different heart model configurations, based on the 70 virtual ventricle shapes as generated in Section 1.1. For each shape, a PK fibre network was constructed, which was then supplemented with the addition of a FT. We obtained six new configurations (Fig 5) where the FT connected into the PPM, the APM or the ventricular free wall (three subtypes) and each of the FTs was created either with a direct connection at the end, or with a delta connection (two subtypes). All the simulations on these configurations were run with a PK system featuring a LBBB and an additional simulation with the healthy Purkinje system without LBBB and FT. In total, this resulted in 8 different configurations per geometrical shape (*i.e*., 560 simulation runs).

Quantification of the influence of the FT on the AP propagation was based on the reduction of the LBBB-induced QRS_d_ prolongation observed between the “LBBB, no FT” and “LBBB with FT”-cases (we denote the difference in QRS_d_ by QRS_Diff_). As each 10 ms increase above the QRS_d_ of 80 ms has been associated with 10% increase in CV mortality risk [18], we quantified the FT-induced reduction of QRS_d_ by 10 ms intervals. Subsequently, we tested whether the total ventricular activation time was reduced to such an extent that QRS_d_ would fall below QRS_d,TH_, such that CRT would not be recommended by international guidelines. Finally, we used the Kolmogorov–Smirnov test to investigate the similarity of the population featuring a LBBB with FT and without FT to the healthy population.

**Fig 6:**
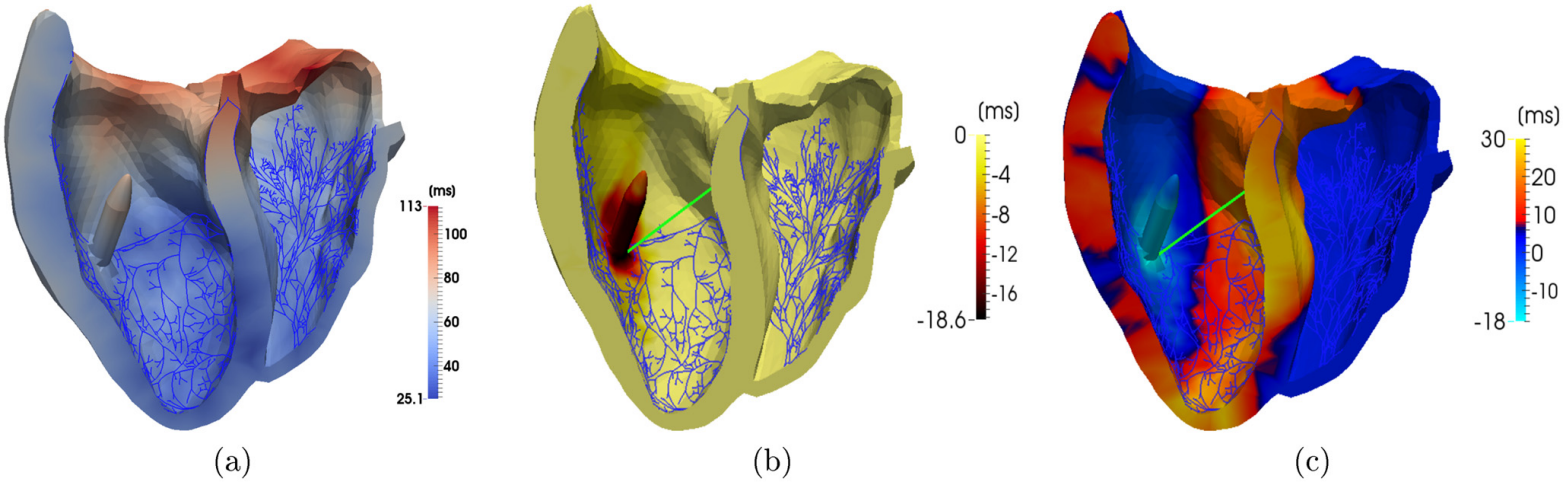
Comparison of the activation pattern with and without false tendon for the same heart. (a) The local activation times for the heart without FTs, (b) subtracted the activation time of the heart with a FT to the ventricular free wall, (c) the same with a left bundle branch block and a FT to the anterior papillary muscle.

For the healthy heart, the experiments revealed a QRS_d_ of 108.5 ± 7.1 ms, which is only slightly affected by a FT. However, the activation time near the FT attachment point decreases locally (Fig 6b). The control cases with a LBBB but without FTs show an increased mean QRS_d_ to 131.9 ± 9.8 ms. This is an expected result since the LBBB disrupts the conduction toward the PK network, delaying the activation until its breakthrough at the septum from the RV.

However, the experiments with a LBBB shows a clear benefit from the presence of a FT.

The simulations including FTs to the ventricular free wall show the shortest mean QRS_d_, while a FT connecting to the PPM gave the only mean QRS durations above the threshold QRS_d,TH_. In general, the direct connection results in shorter QRS_d_ than the delta connection (Table 4), which may be explained by the fact that the electrical impulse propagates partly in the slow conduction myocardium before re-entering the PK fibre network.

**Table 4:** The mean QRS duration for n = 70 geometries and different configurations of the heart model, and the proportion with QRS duration longer than the threshold Q_d,TH_ = 120 ms. (**LBBB**) Left bundle branch block, (**PPM**) posterior papillary muscle, (**APM**) the anterior papillary muscle, and (**VFW**) the ventricular free wall.

	No FT		LBBB and Direct FT to			LBBB and Delta FT to		
QRS Duration	Healthy	LBBB	APM	PPM	VFW	APM	PPM	VFW
Mean [ms]	108.5 ± 7.1	131.9 ± 9.8	108.1 ± 7.5	110.1 ± 6.5	105.8 ± 7.1	116.0 ± 9.6	124.3 ± 11.74	111.5 ± 7.1
>*T*_*th*_ in %(n)	4 (3)	94 (66)	4 (3)	9 (6)	4 (3)	31 (22)	57 (40)	10 (7)

**Fig 7:**
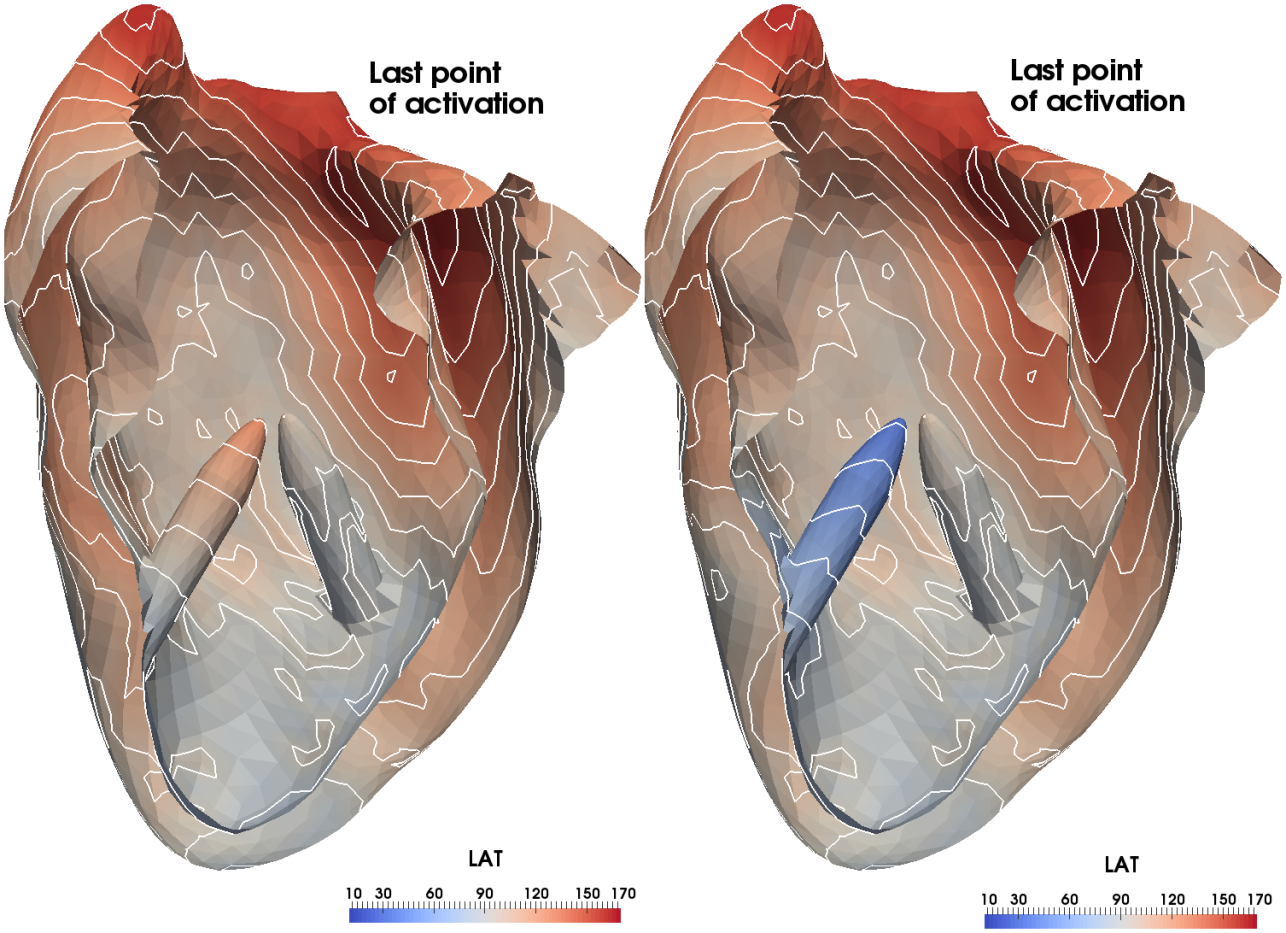
QRS_d_ prolongation. In a few cases the QRS duration may be prolonged by adding a false tendon. In these cases (left without false tendon), the last point activated is still reached by the wave front from the right ventricle, but there is an earlier onset of myocardial activation, here seen in the papillary muscle (right with false tendon).

In most cases the presence of a FT decreased the QRS_d_ by more than 10 ms (Fig 5). Badheka et. al [18] showed that this reduction indicates a more than 10% lower risk of CV mortality. FTs with direct connection showed a QRS_Diff_ of 20 ms or more, suggesting a stronger effect of the alternative conduction pathway produced by FTs of this type. In few cases the FT prolonged the QRS_d_. This might be explained by an earlier onset of the AP in the myocardium due to the FT, but the total activation time is still determined by the AP front originating from the right ventricle (Fig 7).

**Fig 8:**
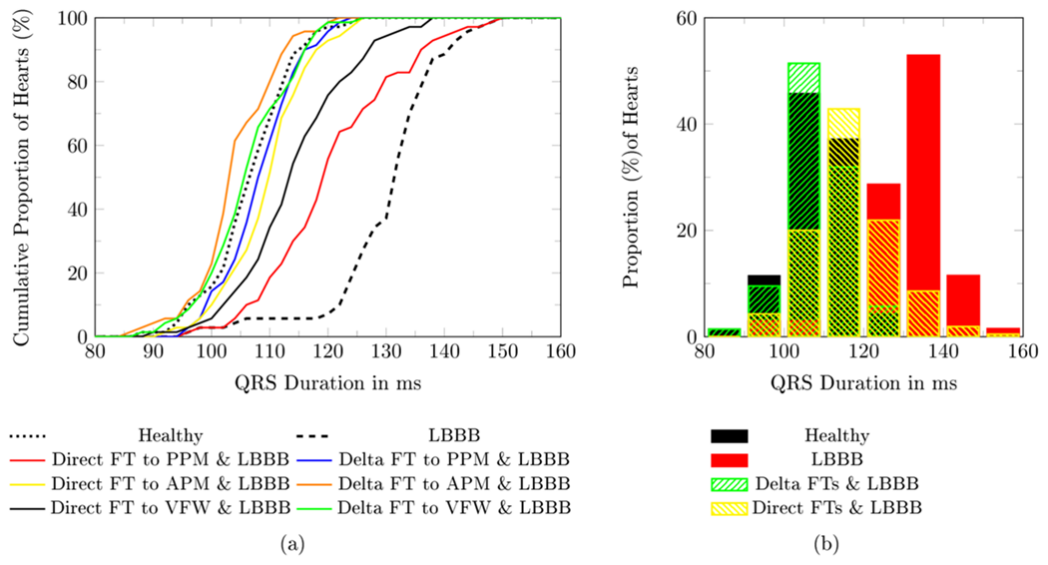
Histogram of the QRS_d_ in the study population (n = 70). (a) Cumulative histogram for all sub groups, (b) Histogram for the healthy, the LBBB, all case with direct connected FT and LBBB and all case with delta connected FT and LBBB. **LBBB** left bundle branch block, **PPM** posterior papillary muscle, **APM** anterior papillary muscle, **VFW** ventricular free wall, **FT** false tendon.

Evaluating the QRS_d_ with respect to the CRT treatment recommendation showed a benefit from the FTs (Table 4 and Fig 8b). To see this we compare subjects with LBBB and no FT to subjects with LBBB and FT. In case of LBBB, the majority of the subjects, 94%(n = 66), have a QRS_d_ longer then the threshold QRS_d,TH_. For a LBBB in conjunction with a FT and delta connection, only 33%(n = 69) of the subjects have a QRS_d_ longer than QRS_d,TH_. With a direct connection of the FT, even fewer subjects remain over the threshold, 8%(n = 16).

Subsequently, we compared the differences in the cumulative distributions of the QRS_d_ (Fig 8) with a two sample Kolmogorov-–Smirnov test, which confirmed with a significance of *α* = 0.01 the similarity of the healthy heart with that featuring a FT with direct connection and a LBBB. In contrast, when comparing the healthy heart with a pathological heart due to a LBBB, the Kolmogorov–Smirnov test is still rejected at the much smaller *α* = 0.0001.

### 2.3 Synchrony in left and right ventricular activation

**Fig 9:**
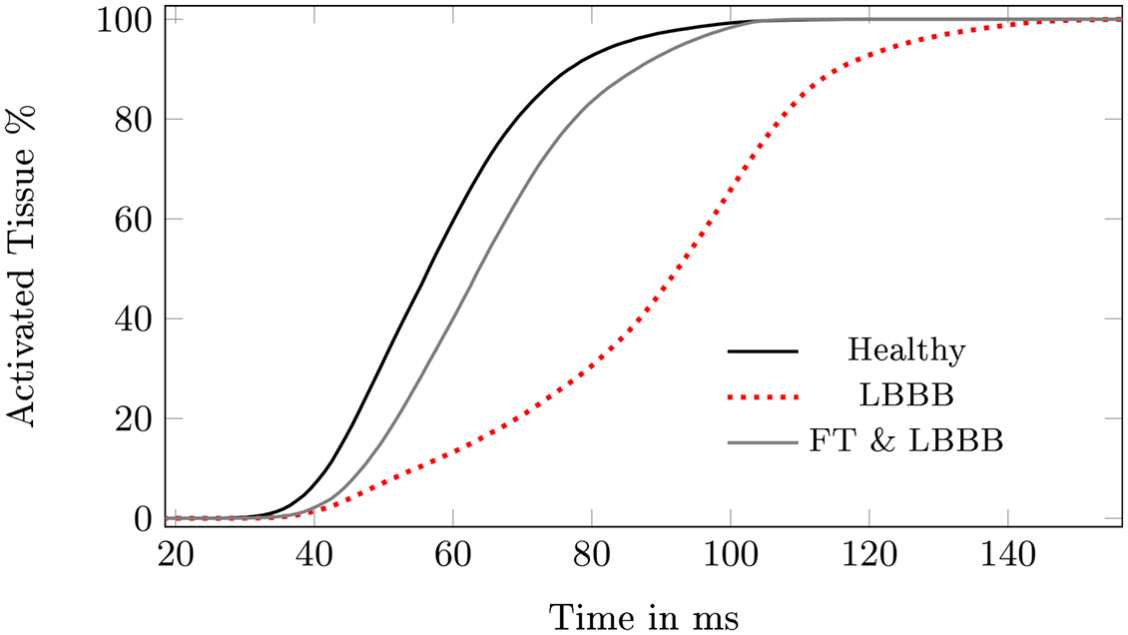
The cumulative percentage of myocardial tissue activated over time. Activated tissue for the healthy heart, the same heart with left bundle branch block (LBBB), for a heart with a direct connected false tendon (FT) to the ventricular free wall and LBBB. The heart in healthy condition and the case of a LBBB with FT are similar, while in case of a LBBB the slope is smaller.

In this experiment, we examined the synchronous activation of the LV and RV for a healthy heart, a heart with a LBBB, and a heart with a LBBB and a FT. To do so, we employed a cumulative histogram of activated tissue (Fig 9).

**Fig 10:**
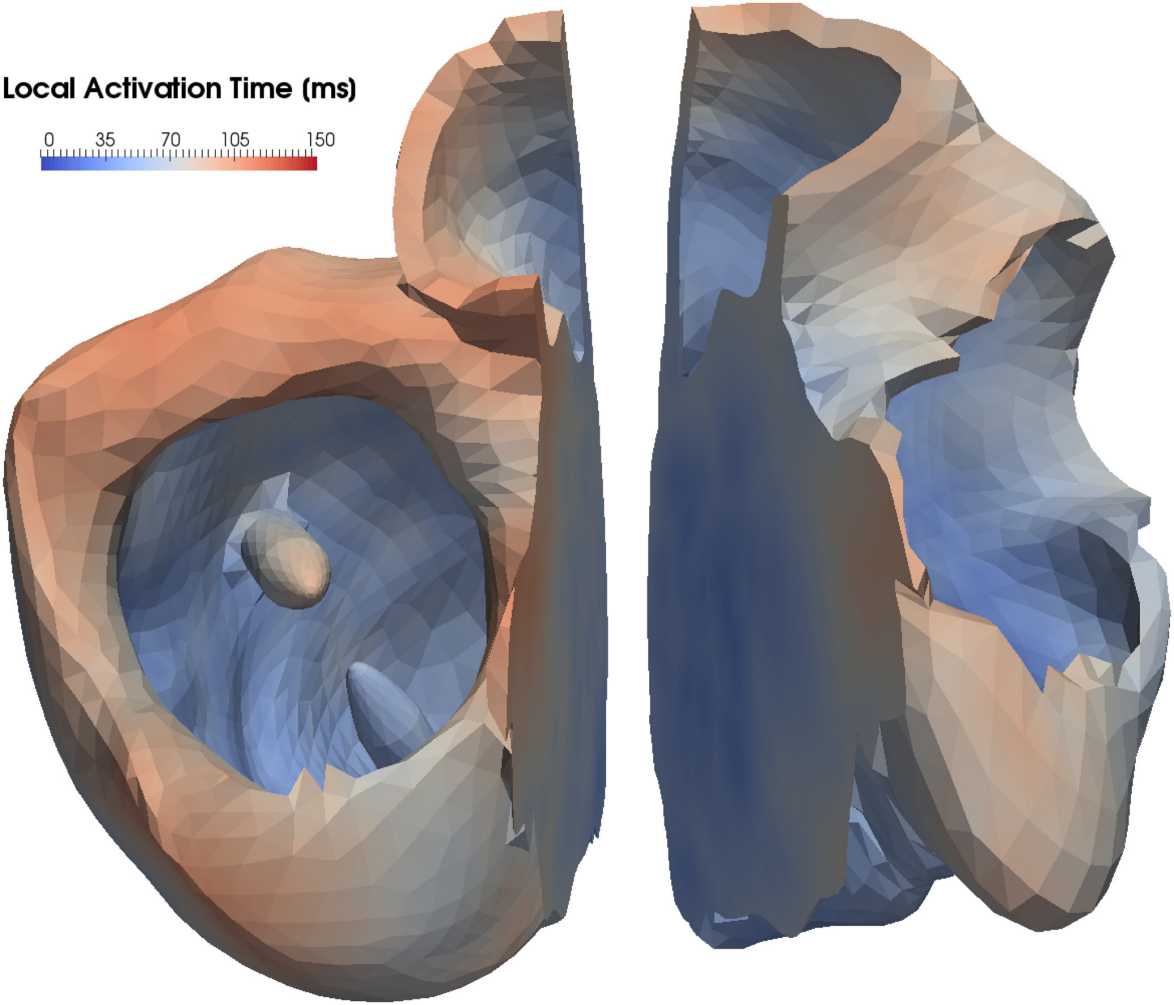
Manual division of the myocardium by a plane in left and right ventricle. This division has been used to estimate the activation time in the different ventricles.

To measure the synchronicity of the activation, we manually divided the myocardium in the LV and RV by a planar boundary in the middle of the septum (Fig 10). Subsequently, we extracted the first activation time in the LV, as well as the time from the onset of the activation in the His bundle until the last point in the LV becomes activated. For the RV, the same measures were calculated. Finally, we calculated the time difference between the last point of activation in the RV and the last point of activation in the LV to evaluate the (dys)-synchrony of the cardiac activation.

The results (Table 5 and Fig 9) indicate that the dys-synchronous activation of the LV and RV in a LBBB is compensated for by the presence of the FT. The initial activation of the LV in the presence of a LBBB but without a FT occurs 12 ms later compared to the healthy heart. In the presence of a FT, however, this difference is reduced to 4 ms only. The third row in Table 5 shows that in the healthy case, the LV was fully activated after 97 ms, while in the case with a conduction block, 125 ms elapsed before full activation. This difference in activation time can be overcome by a FT, whereby the LV is activated 11 ms earlier. In the presence of a FT, the activation in the LV proceeds within the same period as in the healthy case, but from a different starting point (Fig 6a and 6b). The AP travels from the FT to the PM toward the septum and basal area. The activation in the direction of the basal area is comparable to the healthy case, but the activation of the apex and the septal region occurs later. Thus, even when the total activation times are within a healthy range, the contraction pattern needs to be evaluated before we can judge the efficiency of the contraction.

**Table 5:** For a healthy heart, the same heart with left bundle branch block (LBBB) and the same heart with direct connected false tendon (FT) to the ventricular free wall and LBBB times are compared. The time the action potential needs from the activation of the Bundle of His to the first activation in the left ventricle (LV) and right ventricle (RV) are compared, and the time elapsing from the first activation in the LV/RV to the last activation in the LV/RV. This reveals that in case of LBBB the RV and LV are not synchronous in activation, but a FT reinstates part of the synchrony.

Activation time [ms]	Healthy	LBBB	FT&LBBB
First LV	23	35	27
First RV	26	26	26
Total LV	97	125	86
Total RV	73	98	86

It is worth noting that the RV is affected in the same way by the LBBB and the FT. The main effect is the missing activation in the septal area due to the failure of activation from the LV.

### 2.4 The effect of heart size and shape on the FT-induced QRS_d_ reduction

So far this study has looked at different types of FT across a virtual population of heart shapes to evaluate the possible benefit of a FT in case of a LBBB. An interesting additional question is, what influence do the shape of the heart, the size of the heart, or the FT configuration have individually on the ventricular activation time or when compared against each other? Therefore, we performed an additional study consisting of three different sample populations to characterise the influence of each of these three factors independently from each other. For simplicity, we define the size of the heart as the LV length.

The first population (P1) was generated by choosing randomly four heart shapes of the 70 hearts in section 2.2 and then scaling them to have LV length between 70–90 mm with 2 mm intervals. For each of the 40 hearts, a Purkinje system with and without FTs has been generated, where the attachment point of the FT to the APM/PPM was fixed on the top of the papillary muscle and the FT to the ventricular free wall was always attached the same point on the ventricular free wall. This population was used to characterise the effect of size on ventricular activation time.

The second population (P2) was generated to characterise the effect of heart shape. It was built using all 70 shapes from section 2.2, but rescaled to a fixed LV length. For each shape a Purkinje system was generated with all types of FT, where the attachment points were fixed to match those used in P1.

In the third population (P3), the FT attachment point on the ventricular free wall was varied. Therefore, the four heart shapes previously used to generate P1 were scaled to 80 mm LV length, and a Purkinje system without FT was generated. These four Purkinje networks were than used as a basis for Purkinje networks with FT connecting to the ventricular free wall. Randomly chosen attachment locations of the FT in the ventricular free wall were selected to generate 50 different Purkinje networks per shape.

**Fig 11:**
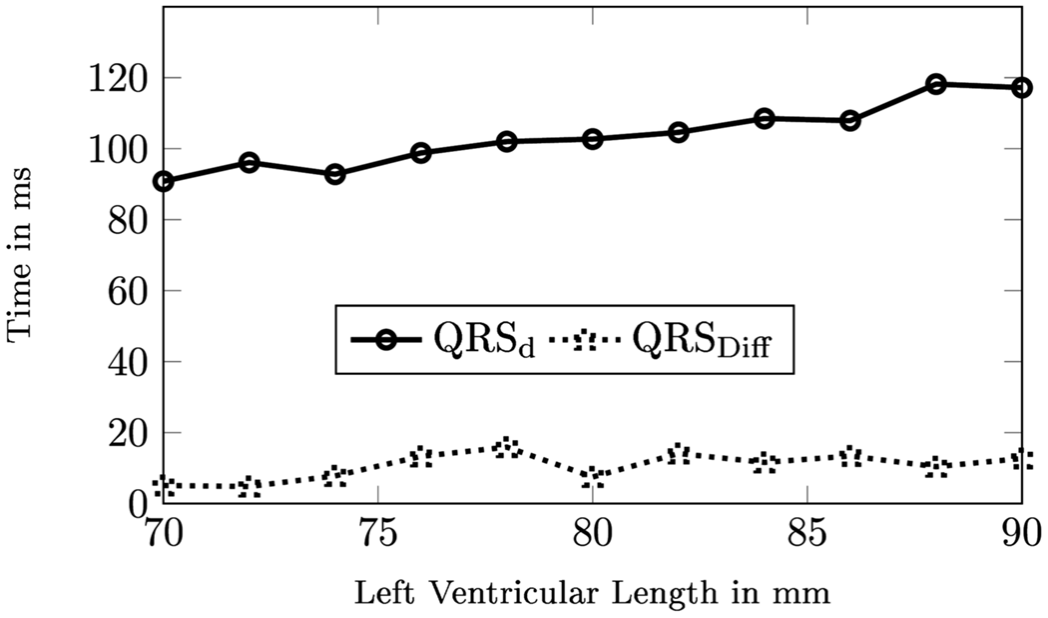
QRS Duration as a Function of Left Ventricular Length. The QRS duration (QRS_d_) for different sizes of the left ventricle, were a LBBB and a false tendon to the anterior papillary muscle is present. The improvement between the “LBBB, no FT” and “LBBB with FT”-case is given by QRS_Diff_.

By performing simulations using the Eikonal model, we can study how the ventricular activation time is affected by the LV size in P1. To quantify this, we recall the definition of QRS_Diff_ which is the change in ventricular activation time observed between the “LBBB, no FT” and “LBBB with FT”-cases (Table 6). As one would expect, the ventricular activation time has a general tendency to increase as the LV length increases, as shown for one case in Fig 11 (top). Somewhat surprisingly, the suspicion that QRS_Diff_ would show the same behaviour turns out not to hold in this particular case. Initially increasing with LV size, QRS_Diff_ seems to reach a particular value and then plateau out (Fig 11, bottom). This may indicate that the size of the LV has relatively little influence on the reduction in ventricular activation time, or that the effect will at least be less pronounced for small hearts.

**Table 6:** Mean and Standard Deviation of the QRS_Diff_ [ms] in the Different Populations.

	PPM-Delta	PPM-Direct	APM-Delta	APM-Direct	VFW-Delta	VFW-Direct
P1: Case 1	4.69 ± 5.42	7.32 ± 4.46	4.16 ± 4.76	10.62 ± 3.60	8.46 ± 4.05	9.96 ± 3.91
P1: Case 2	9.41 ± 4.55	17.38 ± 4.26	17.00 ± 5.04	24.74 ± 3.55	19.22 ± 3.29	23.86 ± 2.23
P1: Case 3	8.95 ± 5.92	22.08 ± 4.87	11.55 ± 6.66	23.80 ± 5.33	21.30 ± 4.00	27.76 ± 3.99
P1: Case 4	10.83 ± 5.28	19.74 ± 3.63	8.96 ± 5.76	20.85 ± 2.83	21.24 ± 3.11	24.96 ± 2.53
P2	8.49 ± 8.64	18.96 ± 8.91	14.38 ± 8.80	21.23 ± 9.02	18.42 ± 8.23	23.73 ± 8.44
P3: Case 1	N.A.	N.A.	N.A.	N.A.	14.02 ± 0.00	14.02 ± 0.00
P3: Case 2	N.A.	N.A.	N.A.	N.A.	17.30 ± 2.36	20.26 ± 1.59
P3: Case 3	N.A.	N.A.	N.A.	N.A.	19.47 ± 2.68	23.86 ± 2.47
P3: Case 4	N.A.	N.A.	N.A.	N.A.	14.80 ± 2.44	15.91 ± 0.12

^PPM^ Posterior Papillary Muscle

^APM^ Anterior Papillary Muscle

^VFW^ Ventricular Free Wall

^N.A.^ Experiement Not Conducted

To compare the change in QRS_Diff_ across populations rather than just between individuals, we report the interquartile range (IQR) of variation in the QRS_Diff_ measure over P1, P2, and P3 respectively in Table 7. Applied to QRS_Diff_ the IQR gives the range of improvement in which 50% of the measured data lie. As such the IQR quantifies the range of data distribution.

**Table 7:** Interquartile Range in [ms] of the QRS_d_ Improvement in each Population and Case.

	P1 (size)				P2 (shape)	P3 (FT configuration)			
	Case 1	Case 2	Case 3	Case 4		Case 1	Case 2	Case 3	Case 4
PPM Delta	9.58	4.67	6.91	6.00	15.20	N.A.	N.A.	N.A.	N.A.
PPM Direct	8.16	6.03	4.57	3.41	9.64	N.A.	N.A.	N.A.	N.A.
APM Delta	6.07	5.57	10.67	10.29	10.51	N.A.	N.A.	N.A.	N.A.
APM Direct	5.68	3.48	5.32	3.72	10.83	N.A.	N.A.	N.A.	N.A.
VFW Delta	7.63	3.20	5.12	4.36	10.97	0.00	3.18	3.67	0.24
VFW Direct	6.50	2.40	4.19	2.32	9.29	0.00	1.61	3.23	0.00

^PPM^ Posterior Papillary Muscle

^APM^ Anterior Papillary Muscle

^VFW^ Ventricular Free Wall

^N.A.^ Experiement Not Conducted

The IQR in P2 is in almost all cases much larger than in P1, with as much as four times the values in P1. This means that the range of improvement is much larger for the shape population compared to the size population. This conclusion is further supported by the fact that the mean QRS_Diff_ varies considerably between subjects in P1 (Table 7). Both observations together are strong indicators that in this model the improvement of the QRS_d_ in LBBB is more sensitive to shape then LV length.

The same analyses can be done between P2 and P3 to compare the sensitivity of shape vs. FT location. This comparison is even more striking, as it can be seen that for certain configurations the observed IQR can vanish, which is also reflected in the smaller standard deviation in P3 (Table 7). At first this appears surprising, however an explanation is given by looking at the activation patterns. If the improvement by inserting a FT does not depend on the FT attachment location, it means that the last part of the heart to get activated is by an action potential arriving from the RV, which should be independent of the FT attachment point.

## 3 Limitations

In this work we chose the Eikonal equation for simulating AP propagation, which provides information on the activation times only. A limitation of this model is that it does not account for ionic currents in ventricular cells, and as such it is not able to simulate charge accumulation or arrhythmia. Even though the Eikonal equation approximates the local activation times very well, the QRS_d_ estimated from an surface ECG can differ from the QRS_d_ measured on the endocardial surface.

Furthermore, the current model does not include multiple FTs in one heart, which are occasionally observed [4], but our model could be extended to cover this eventuality. A further limitation of the current model is that the FT is modelled as a single Purkinje fibre, whereas in the literature it has been observed that a FT can feature also myocardial and fibrous tissue, which could have a contribution in the electrical conduction. Our work constitutes a first step towards more advanced simulations and one that is necessary to establish the importance of FTs in the study of cardiac physiology and function.

The current study does not investigate the exact relationship that the shape of the hearts and the FTs has on the reduction on QRS_d_. Therefore, a parametrisation is needed where each parameter corresponds to clinically intuitive and identifiable features [72]. Such an approach would lend itself to visually assess FT effects on LBBB patients based on imaging data and supplementing the knowledge of the type of FT in question and surface ECG.

Measurements of the action potential in the Purkinje system are very limited and do not include systematic studies of the propagation of the action potential. Moreover, reports of FTs in humans are very few and mostly limited to case studies, which makes it hard to validate our model against experimental data. Nevertheless, the Eikonal equation and the Purkinje network model have been previously shown to reproduce local activation times reasonably accurately [56–58].

## 4 Discussion and Conclusions

This study demonstrates and quantifies for the first time the effect of the presence of the FT in terms of QRS_d_. It has been carried out in anatomically accurate models of the heart in the presence of LBBB, where the FT is assumed to contain fast conducting fibres. This assumption is motivated by a recent observation by Irie et al. [14] and the similarities between Purkinje fibres in the FT and the bundle of His, but lacks extensive clinical evidence. However, our work provides a motivation for gathering further clinical evidence, as this study shows there are two possible ways in which the FT can influence the activation pattern. First, it may lead to an increased CV mortality risk; and second, it may have practical implications on patient management.

The CV mortality risk is largely influenced by QRS_d_ as each 10ms increase above 80ms has been associated with a 10% increase in the CV mortality risk, as shown by Badheka et al. [18]. In our in-silico experiments almost all cases show a QRS_d_ reduction, where the mean reduction was 20 ± 10 ms with a maximum of 45 ms. The shortened QRS_d_ is also reflected in an improved synchronous activation of the LV and RV. Overall this indicates a substantial improvement, but also the treatment recommendation can be affected.

The amount of QRS_d_ reduction reported in this paper highlights the impact of FT on practical patient management. Current guidelines do not recommend CRT for LBBB patients with QRS_d_<120 ms. In our experiments 97% of simulation with no FT present that had QRS_d_>120 ms, after introducing the effect of the FT, experienced a reduction in their QRS_d_ below the patient selection criteria. This effect however is dominantly present with FTs of the direct connection type. Therefore, both the presence of the FT and the structure of their attachment has to be taken into consideration. Overall, a FT seems to impact the QRS_d_ and affect the recommended management of LBBB patients. However, in the same way the presence of a FT can mask the severity of the LBBB.

By looking at the three parameters shape, LV length and FT attachment point, we have learned that the QRS_d_ decrease seems to be most sensitive to shape, whereas size still influences the decrease to certain extent, and the FT location on the ventricular free wall has minor or no influence at all.

As any model, our model has limitations. With respect to our study, there are two important to keep in mind. First, the study was performed with the Eikonal model, which only models wave propagation, but no charge accumulation or specific ion channels. The second is, that the treatment recommendations are with respect to the surface ECG, while our QRS_d_ are obtained within the heart.

Overall, our results demonstrated that in some cases of pathological conduction it is relevant to consider FTs in the electrical activity of the human heart. Future work should investigate FTs in more detail. A first step will be to further develop our model using the monodomain or bidomain equations and to refine the FT anatomical model. Such refined models could then be used to answer the question of how the FT might affect CRT outcome. Finally, the model could be used to begin explaining unusual electrocardiographic observations like inverted T waves.

## Supporting Information

S1 File Example geometries with Simulations Results. (ZIP)

S1 Table Spreadsheet containing simulation results. (XLSX)
